# A Rare First Presentation of Hodgkin's Lymphoma: Symptomatic Pericardial Effusion

**DOI:** 10.7759/cureus.46731

**Published:** 2023-10-09

**Authors:** Rita Matos Sousa, Carolina Nogueira, Maria João Vilela, Fábio Neves Correia, Catarina Silva Araújo, Maria Ribeiro, Carlos Capela

**Affiliations:** 1 Internal Medicine, Hospital de Braga, Braga, PRT; 2 Clinical Sciences, Escola de Medicina da Universidade do Minho, Braga, PRT; 3 Intensive Care Unit, Hospital de Braga, Braga, PRT; 4 Onco-Hematology, Hospital de Braga, Braga, PRT

**Keywords:** classic hodgkin lymphoma, lymphoproliferative malignancy, pericarditis, pericardial diseases, pericardial effusion

## Abstract

Symptomatic pericardial effusion occurring as the initial manifestation of Hodgkin's lymphoma is exceedingly uncommon, and there are limited documented instances in the available literature. Pericardial effusion can present various differential diagnoses, and among these, malignancy is an important yet less frequently encountered cause. A heightened level of suspicion is crucial for establishing an accurate diagnosis, particularly when the patient's clinical course deviates from the anticipated trajectory. Through this case, we aim to emphasize the significance of considering lymphoproliferative diseases as a pertinent possibility in the differential diagnosis of pericardial effusion. Additionally, we underscore the importance of promptly reaching a diagnosis, as it can help prevent severe complications and enhance the patient's prognosis.

## Introduction

Cardiac involvement in Hodgkin's lymphoma (HL) is described in the literature as being infrequent and possibly explained by direct malignant involvement of the pericardium or due to indirect consequences, namely, obstructive, irritative/inflammatory, and immunological, among others [1,2]. Pericardial effusion is present in about 5% of patients with HL; however, it is usually asymptomatic and rarely the initial presentation of HL [3-6].

Here, we report a case of a 28-year-old woman with palpitations, who underwent an echocardiogram that revealed pericardial effusion. She was first diagnosed with pericarditis, which did not respond to appropriate treatment. Later on, due to the persistence of symptoms and pericardial effusion, she underwent further analytical, imagological, and histological work-up that revealed an HL. With this case, we hope to highlight the importance of lymphoproliferative diseases as a relevant differential diagnosis of pericardial effusion and the importance of a quick diagnosis that might prevent serious complications and improve the patient's prognosis.

## Case presentation

A 28-year-old woman with symptoms of palpitations attended a cardiology consultation where a pericardial effusion was identified on the echocardiogram. On physical examination, the cardiologist referred to tachycardia and a pericardial rub on cardiac auscultation. Initial workout detected highly pro-inflammatory markers (C-reactive protein of 170 mg/L and sedimentation velocity of 92 mm/h), negative for auto-immunity markers, an electrocardiogram showing sinus tachycardia, and a normal chest X-ray. With these data, the cardiologist assumed the diagnosis of idiopathic pericarditis. The patient was started on non-steroidal inflammatory drugs and colchicine and was reassessed one month later. At that time, the pericardial effusion had grown, and the patient was presenting signs of respiratory difficulties for small efforts, tachycardia, asthenia, and dry cough associated with a pleuritic thoracalgia at the lower half of the left hemithorax. At no point the patient presented fever, weight loss, night sweats, anorexia, changes in the gastrointestinal tract, arthralgia, or skin lesions. The inflammatory markers remained high. An ultrasound was performed that revealed abdominal and left supra-clavicular adenopathies with reactive characteristics.

**Figure 1:**
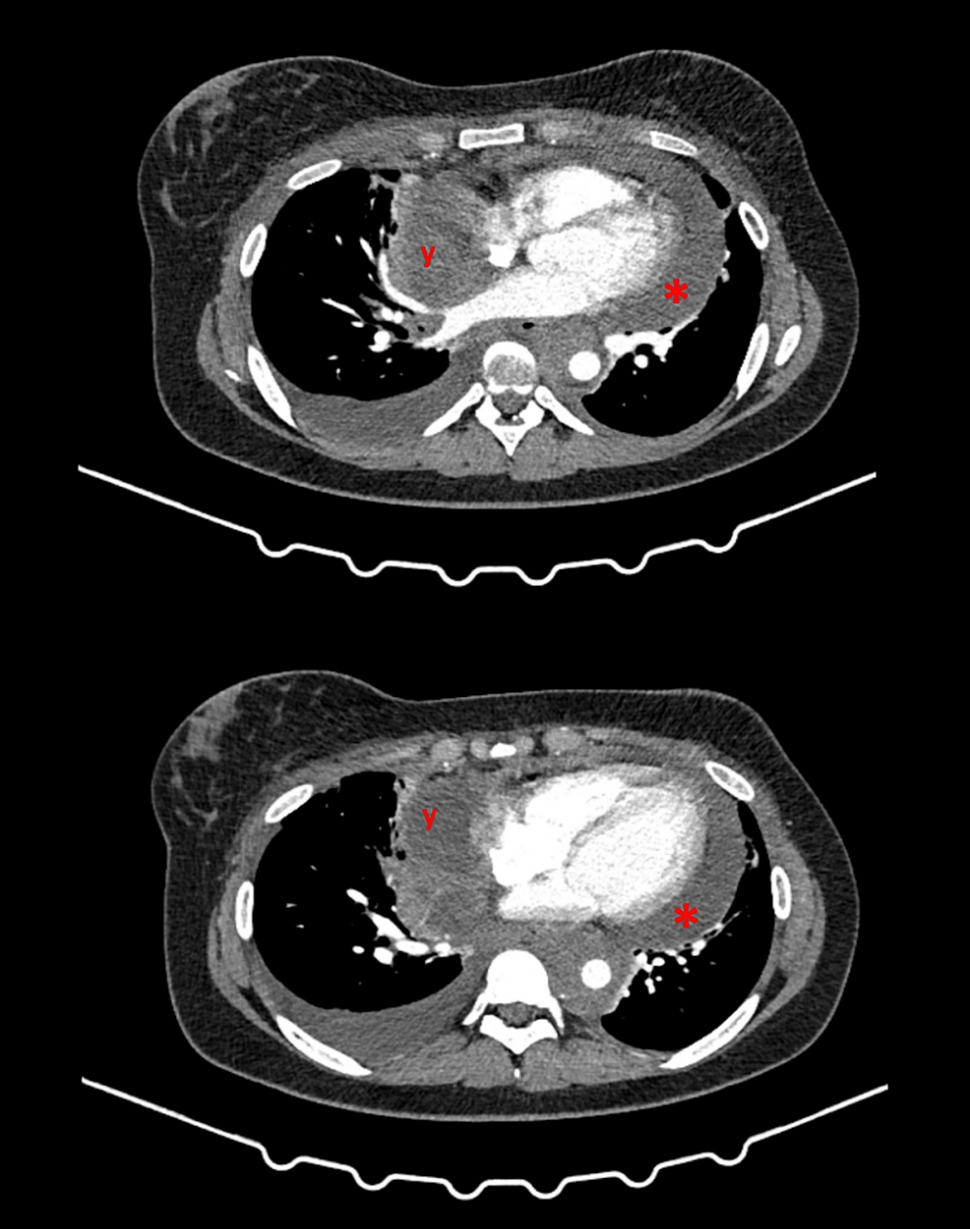
CT scan showing pericardial effusion (*) and a large mediastinal mass (y).

She was sent to the emergency department for further evaluation. A transthoracic echocardiogram was performed revealing a large volume pericardial effusion, without signs of tamponade. A pericardiocentesis was performed despite the absence of tamponade to help with the diagnosis of the pericardial effusion' cause and to improve the patient's symptoms. We drained 700 cc of hematic fluid, with the improvement of symptoms. Laboratory examination revealed anemia with a hemoglobin of 10.2 g/dL, a white blood count of 11.9 with 83.5% neutrophils, a platelet count of 608, and an albumin level of 3.5 d/dL. The pericardial fluid did not present any malignant cells and had oxidative characteristics. A computed tomography scan of the thorax, abdomen, and pelvis (Figure 1) revealed a large mediastinal mass of probable lymphomatous nature of infiltrative characteristics, which occupies the prevascular, pretracheal, pre- and infracarinal spaces, and the aortopulmonary window, with wavy contours, extending in contiguity to the right pulmonary hilum. It invaded the right anterior chest wall and the small and large pectoral muscles, with lobulation of its contour, and destroyed the anterior arch of the second right rib with a primitive component. There were multiple adenopathies at levels I, II, and III in the right armpit and in the supra and infraclavicular chains bilaterally and an adenopathic conglomerate in the right anterior paracardiac space and the internal mammary chains. There was a circumferential pericardial effusion of medium volume, with a pericardium thickening zone in its right anterior aspect, for probable involvement by contiguity. It involved circumferentially the mouth of the right pulmonary veins and superior vena cava.

**Figure 2:**
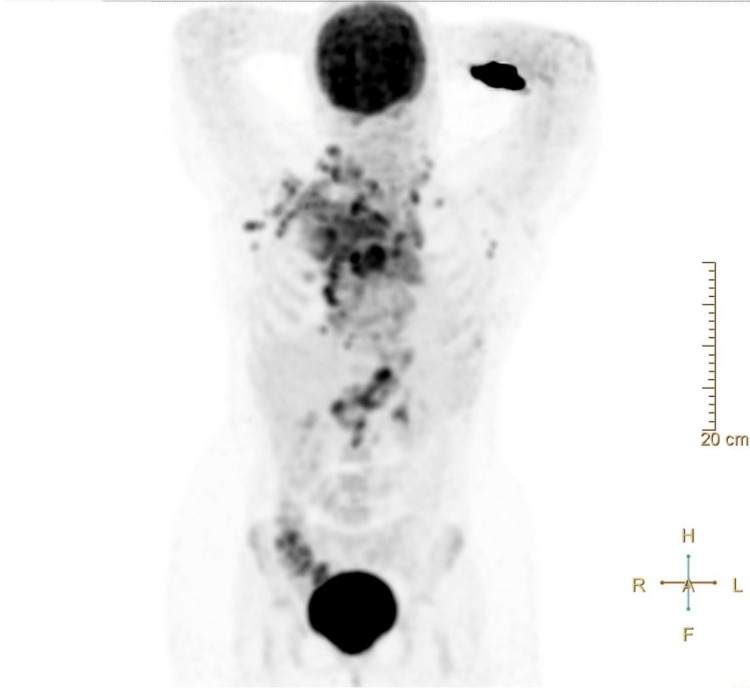
PET scan showing a large mediastinal mass; pleural, pericardial, bone, and muscular infiltration; and hypermetabolic infra and supra diaphragmatic adenopathies.

At this point, the patient was admitted to a high-vigilance ward due to a probable lymphoproliferative disease with a high risk for compressive symptoms and complications due to its size and location, and a large volume pericardial effusion with minor pleural effusion. During her stay in the ward, the patient underwent a positron emission tomography scan (Figure 2) that confirmed the presence of a large mediastinal mass with intense glycolytic metabolism. It also showed plural, pericardial, bone, and muscular infiltration, and hypermetabolic infra and supra diaphragmatic adenopathies. The bone marrow biopsy was free of involvement.

The biopsy of the mediastinal mass confirmed the diagnosis of nodular sclerosis classic Hodgkin's lymphoma, stage IV-A, with an International Prognostic Score of 4. At this point, the patient was stable and started on directed treatment with steroids and chemotherapy. The patient was referred to the hematology-oncology specialty for treatment and follow-up. After six months of treatment, the patient showed significant improvement in symptoms and a reduction of the pericardial effusion. The mediastinal mass was also significantly smaller, showing adequate response to the treatment.

## Discussion

Pericardial effusion is an important finding that has many differential diagnoses. The presentation of pericardial effusion can have a broad spectrum of symptoms and can even be asymptomatic. When approaching a pericardial effusion, some causes must come to mind: inflammatory, such as infection, auto-immune, uremic pericarditis, or even drugs; and non-inflammatory, such as neoplastic, metabolic, traumatic causes, or reduction of lymphatic drainage [7-10]. The most common cause of pericardial effusion is idiopathic pericarditis and is usually presumed to be post-viral. In this case, the first diagnosis to be made was idiopathic pericarditis due to the age and clinical presentation of the patient, with only palpitations and increased inflammatory markers. Other infections such as bacterial or fungal infections are usually more symptomatic. Another important diagnosis, especially in recurrent pericarditis is tuberculosis infection and it must be ruled out by testing for tubercle bacilli in the pericardial liquid. Malignancy must also be suspected in cases of pericardial effusion recurrence, even though malignant cells might not be present in the pericardial fluid, as was the case of this patient. Nevertheless, the cancers that are more associated with pericardial effusion are lung cancer, breast cancer, melanoma, and lymphoma, so diagnostic tests must be made to exclude these diseases [11-13].

The most common mechanism for pericardial effusion of malignant etiology is the involvement of the lymphatic and venous drainage of the pericardium. In patients with already known malignancy, the most probable causes for pericardial effusion are malignant pericardial effusion, radiation-induced pericarditis, drug-induced pericarditis, and idiopathic pericarditis [13]. Specifically in HL, pericardial effusion is more associated with the classical variant and its typical presentation with a bulky mediastinal mass that can compromise the lymphatic and venous drainage. In this case, we believe this was the most probable mechanism. Regarding the other mechanisms, the patient was still unknown to have a malignant disease and was therefore naïve to any treatment, making the radiation-induced and drug-induced pericarditis less likely. Also, as previously discussed, idiopathic pericarditis was the first assumption diagnosis to be made but the lack of response to the appropriate treatment also discarded this option.

HL, such as other lymphoproliferative diseases, might have an insidious clinical presentation with constitutional symptoms such as weight loss, chills, night sweats, and fever. It is important to highlight that there are two peaks for the diagnosis of HL: the first between ages 15 and 40 and the second in people over 60 years of age. Despite being highly associated with malignant diseases, constitutional symptoms are not the only possible presentation. Regarding HL, patients can also present with lymphadenopathy, hepatic and spleen enlargement, and pruritus [14,15]. However, in the literature, there are some reports of atypical and rare first clinical presentations, such as the one we describe here [4,6,16-18]. In fact, pericardial effusion is a rare finding, accounting for about 5-6% of the patients diagnosed with HL. Most pericardial involvement in HL is silent and is an imagological finding, usually of small volume, and rarely is the first presentation of the disease. When pericardial effusion is presented with symptoms, the most common are dyspnea, anterior thoracalgia, pleuritic thoracalgia, and peripheral edema [13].

Finally, it is important to enhance that given this rare presentation of HL, the final diagnosis was delayed, which may have brought serious risk and prognosis impact to this patient. Although the literature is scarce about the effect of diagnosis delay in HL, Norum states the major problem might be the aggressiveness of the tumor. Also, other non-directly related complications, such as superior vena cava syndrome, might have occurred in the case of this patient [19].

## Conclusions

Pericardial effusion as the presentation of HL is rare and its management is challenging. A wrong diagnosis, such as idiopathic pericarditis, can delay the final diagnosis and its treatment and allow for serious complications. Malignant causes for pericardial effusion must be present in the differential diagnosis and excluded. In this case, we intend to highlight that a common diagnosis, although more probable, must not overcome the need for a high suspicion reasoning when the patient fails to meet the expected clinical evolution.
